# Gut microbiota-derived tryptophan metabolites and inflammatory factors enhance prediction of early neurological deterioration after intravenous thrombolysis in stroke patients: a comparative model study

**DOI:** 10.3389/fneur.2026.1934189

**Published:** 2026-10-20

**Authors:** Wei Li, Zhiyan Feng, Chunrong Zeng, Zhimei Cao, Yunfei Bai, Honglei Wang, Jiagui Huang

**Affiliations:** 1 Medical Imaging Center, Second People's Hospital of Yibin City-West China Yibin Hospital, Sichuan University, Yibin, China; 2 Department of Neurology, Second People’s Hospital of Yibin City-West China Yibin Hospital, Sichuan University, Yibin, China

**Keywords:** early neurological deterioration, ischemic stroke, nomogram, thrombolysis, tryptophan

## Abstract

**Background and purpose:**

Early neurological deterioration (END) after stroke thrombolysis predicts poor outcomes. This study developed predictive nomograms that incorporate gut-derived tryptophan metabolites and inflammatory markers to predict END.

**Methods:**

We evaluated demographic, clinical, and laboratory variables, including gut-derived tryptophan metabolites and inflammatory markers. A multivariable logistic regression model was developed to predict END and presented as a nomogram. Model performance was assessed using variance inflation factors, ROC analysis, calibration curves, and decision curve analysis. Net Reclassification Improvement and Integrated Discrimination Improvement were used to compare models.

**Results:**

In this study, END occurred in 11.26% of stroke patients and was independently associated with systolic blood pressure (SBP), fasting blood glucose, and serum levels of indole-3-propionic acid (IPA), indole-3-lactic acid (ILA), and interferon γ (IFN γ). Two nomogram models were developed to predict END: one based on conventional risk factors (SBP and glucose) and another incorporating emerging biomarkers (SBP, glucose, ILA, IPA, and IFN γ). The models achieved AUC scores of 0.719 (95% CI 0.658–0.780) and 0.909 (95% CI 0.879–0.939), respectively. Comparing the models showed a categorical Net Reclassification Improvement of 0.601 (*p* = 0.000) and an Integrated Discrimination Improvement of 0.197 (*p* = 0.000).

**Conclusion:**

Incorporating gut microbiota-derived tryptophan metabolites and inflammatory factors improves prediction of END among thrombolyzed stroke patients.

## 1 Introduction

Acute ischemic stroke (AIS), resulting from blocked brain vessels, leads to tissue damage and is a major global health burden (1). Timely reperfusion is key, with intravenous thrombolysis being a frontline treatment to dissolve clots and restore blood flow. However, not all patients benefit—many experience early neurological deterioration (END), which predicts poor long-term outcomes (2, 3). Identifying high-risk patients before or after intravenous thrombolysis is crucial, as it enables closer monitoring and personalized care that may improve recovery. This highlights the need to explore reliable predictive models of END following thrombolysis in stroke patients.

In AIS patients receiving intravenous thrombolysis, END incidence ranges from 5 to 40%, depending on the definition of the worsening time window and the stroke severity scale used (2). Emergency green channels for stroke patients have increased intravenous thrombolysis rates, warranting closer monitoring of END. Multiple methods, including stroke prognostic scores and machine learning algorithms, have been developed to forecast clinical outcomes in thrombolyzed stroke patients (4). According to Fang et al., a higher NIHSS score, large artery atherosclerosis, and prior diabetes independently predicted END in AIS patients receiving intravenous thrombolysis (1). Tian et al. identified the neutrophil-to-lymphocyte ratio, mean platelet volume, body mass index, and atrial fibrillation as strong predictors of END (3). Wei et al. constructed dynamic nomograms using real-world multidimensional variables to estimate END risk (5). However, existing models rely on retrospective data and conventional risk factors, often with limited sample sizes, and their relevance is diminishing as medical advances emerge. To improve END prediction, future studies should collect large-scale prospective samples and integrate novel stroke prognostic biomarkers into the modeling framework.

Emerging evidence indicates that gut microbiota affects AIS via the gut-brain axis, influencing neuroinflammatory responses, behavioral outcomes, and cognitive function (6). Conversely, patients with unfavorable recovery following AIS presented specific gut microbiota dysbiosis (7). Emerging biomarkers, including gut microbiota-derived tryptophan metabolites and inflammatory markers, offer new predictive potential (8, 9). Integrating these novel factors into updated models is urgently needed to improve prognostic accuracy.

This study aimed to develop and rigorously validate a comprehensive predictive model by integrating conventional risk factors with emerging biomarkers, including gut microbiota-derived tryptophan metabolites and inflammatory factors. The performance of models with and without these novel markers was systematically compared. We aimed to develop a robust, practical model for early detection of patients at high risk of END to enable personalized interventions.

## 2 Materials and methods

### 2.1 Patients

This prospective observational study adheres to the STROBE guidelines (10). The study included adult patients presenting with AIS and receiving intravenous thrombolysis (either tenecteplase or alteplase) within 4.5 h of symptom onset. From October 2021 to February 2026, participants were enrolled at Second People’s Hospital of Yibin City-West China Yibin Hospital, Sichuan University. Diagnosis of AIS was established following the 2018 Chinese clinical guidelines for the management of AIS. Eligible patients met the following inclusion criteria: (1) aged 18 years or older; (2) prior stroke modified Rankin Scale (mRS) score of 0 or 1; (3) AIS confirmed by neuroimaging, typically using non-contrast computed tomography and computed tomography perfusion in the setting of neurological deficits.

Key exclusion criteria comprised: (1) rapid symptom improvement as assessed by the treating neurologist; (2) diagnosis of inflammatory bowel disease, irritable bowel syndrome, prior gastrointestinal surgery, hematopoietic disorders, or autoimmune diseases; (3) acute systemic inflammatory illness within 1 month before enrollment; (4) use of antibiotics or probiotics for >3 consecutive days in the preceding 3 months; (5) life expectancy <1 year due to terminal illness; and (6) history or imaging findings indicative of subarachnoid hemorrhage, cerebral arteriovenous malformation, intracranial aneurysm, or neoplastic brain lesions.

### 2.2 Demographic and laboratory data collection

Demographic and clinical laboratory data were obtained from the hospital’s electronic health records. These included: (1) basic characteristics and pre-admission clinical course: sex, age, smoking status, history of ischemic heart disease, hypertension, atrial fibrillation, diabetes mellitus, prior stroke/transient ischemic attack, pre-admission medications, blood pressure within 24 h, and body mass index; (2) stroke-specific details: onset-to-door time, onset-to-thrombolysis time, NIHSS scores at admission and at 24 h, TOAST classification, occlusion site, thrombolytic agent used, whether endovascular thrombectomy was performed, intracranial hemorrhage within 36 h, and moderate-to-severe systemic bleeding events; (3) laboratory parameters: blood glucose, a complete blood count with differential, hemoglobin, platelet count, lipid profile, uric acid, albumin, creatinine, and homocysteine. Blood pressure variables were derived from measurements obtained before the onset of END. The mean systolic blood pressure (SBP) was computed using all available readings from the start of thrombolysis through the last full NIHSS assessment before END occurred. To evaluate relative hyperglycemia, we calculated the stress hyperglycemia index, defined as fasting plasma glucose (mmol/L) divided by glycated hemoglobin (%) (GAR).

### 2.3 Sample size justification

Sample size determination for the clinical prediction model was justified using the widely accepted 10 Events Per Variable criterion, which ensures adequate statistical power (11). Assuming an anticipated END incidence of 20% and up to 8 candidate predictors in the final model, a minimum of 400 participants was initially required. However, given the observed 11.26% event rate in the final cohort, the 666-patient sample yielded 75 events. This resulted in an events-per-variable ratio of 15, which remains sufficient for model stability.

### 2.4 Assessment of serum gut-derived tryptophan metabolites and inflammatory markers

Venous blood was collected from the antecubital fossa pre-thrombolysis and 24 h post-thrombolysis. Serum was obtained by centrifugation for 15 min at 3000 rpm under refrigeration (4 °C) within 2 h, aliquoted, and archived at −80 °C for later assays. Serum concentrations of indole-3-lactic acid (ILA), trimethylamine N-oxide (TMAO), indole-3-propionic acid (IPA), matrix metalloproteinase-9 (MMP9), lipopolysaccharide (LPS), and interferon γ (IFN γ) were measured prior to thrombolysis using commercially available quantitative ELISA kits (Jiangsu Meimian Industrial Co., Ltd., China) according to the manufacturer’s protocols. The lower limits of quantification were 1 ng/L for ILA, 2 pmol/L for IPA, 20 ng/mL for TMAO, 45 μg/L for MMP9, 20 ng/L for LPS, and 6.25 pg./mL for IFN γ; no samples were below the detection limit. All samples were processed and assayed in blinded batches, with each measured in duplicate and the mean value used for analysis.

### 2.5 Outcome evaluation

END was established as a sustained increase in NIHSS score of at least 4 points within 24 h after admission. Functional outcome was evaluated based on neurological condition at the 90-day mRS. Consistent with prior research, a score of 0 to 2 on the mRS was categorized as a positive outcome, and scores from 3 to 6 as an unfavorable outcome.

Symptomatic intracranial hemorrhage (sICH) was identified based on any hemorrhage-related neurological worsening, indicated by an NIHSS score rise of 4 or more points or a fatal event. Asymptomatic ICH was defined as any hemorrhagic lesion on follow-up imaging without clinical worsening, including intraparenchymal hemorrhage, hemorrhagic infarction, or remote hemorrhage. Bleeding events of moderate-to-severe were defined based on the GUSTO trial criteria. All assessors involved in scoring underwent standardized training and certification and remained blinded to clinical information throughout the study.

### 2.6 Statistical analysis

All statistical evaluations were conducted using the R statistical environment (version 4.4.1). The distribution of continuous variables was assessed with the Kolmogorov–Smirnov normality test. Variables with a normal distribution and equal variance are presented as mean ± standard deviation (SD) and analyzed using independent-samples *t*-tests or ANOVA. Variables that do not follow a normal distribution are summarized as median (interquartile range, IQR) and compared with the Mann–Whitney U test. Categorical data are presented as frequencies (percentages, %) and group differences are assessed by chi-square or Fisher’s exact tests, as appropriate. Missing values were handled using multiple imputation techniques with the MICE and rms packages in R. Ten imputed datasets were generated under the missing-at-random assumption, with continuous variables imputed via predictive mean matching and binary variables via logistic regression. Supplementary Table S1 shows the missing data proportion for each variable.

To develop a predictive model for END, we first performed univariate logistic regression to assess the association of each candidate variable with END occurrence. Variables with a *p*-value below 0.2, selected on the basis of clinical importance and prior literature, were subsequently entered into a multivariate logistic regression analysis. The independent risk factors identified through this process were then used to build the final prediction model. The identified variables were subsequently integrated into a logistic regression analysis. The resulting model was visualized as a nomogram using the “rms” and “nomogram” packages in R. Model development, evaluation, and reporting adhered to the TRIPOD guidelines for prognostic studies.

Multicollinearity among the independent predictors was assessed by examining variance inflation factors (VIF). A VIF value below 5 indicates no substantial multicollinearity. The prediction model underwent internal evaluation across three dimensions: discrimination, accuracy, and clinical usefulness. Discrimination was evaluated by analyzing receiver operating characteristic (ROC) curves and calculating the corresponding area under the curve (AUC). An AUC > 0.7 was considered indicative of moderate to acceptable discrimination. Calibration was assessed with the Hosmer–Lemeshow goodness-of-fit test and visualized with calibration curves. Overall performance was assessed using the Brier score, which calculates the average squared error between predicted probabilities and actual outcomes. Clinical usefulness was further examined using decision curve analysis (DCA). Consistent performance across all evaluation metrics confirmed the successful development of the prediction model.

Model comparison was performed using two metrics: the Net Reclassification Improvement (NRI) for evaluating risk category assignment, and the Integrated Discrimination Improvement (IDI) for assessing overall discrimination. Statistical significance for the NRI and IDI was evaluated using the Bootstrap method with 1,000 replicates to estimate 95% confidence intervals (CI) and *p*-values. Bootstrap resampling was also used to internally validate the prediction model, ensuring a robust assessment of its performance and reliability. Statistical significance was defined as a *p*-value less than 0.05.

## 3 Results

### 3.1 Clinical characteristics of AIS patients in original cohort

Between October 2021 and February 2026, 757 patients received intravenous thrombolysis in our departments. After exclusions—81 treated with urokinase, 4 with stroke mimics, and 6 lost to follow-up—the final analytical dataset comprised 666 patients. Study participants had a median age of 70 years (IQR 60–78), and 36.64% were female. END occurred in 75 patients (11.26%). Compared with those without END, patients with END had significantly higher SBP post-thrombolysis, elevated fasting blood glucose, increased serum uric acid, a higher incidence of sICH, and poorer 3-month neurological outcomes (all *p* < 0.05; Table 1).

**Table 1:** Comparisons of baseline characteristics between participants with and without END.

Variables	Total (*n* = 666)	No END (*n* = 591)	END (*n* = 75)	*p* value
Age, years, median, (IQR)	70 (60–78)	70 (59–77)	72 (61–79)	0.118
Female sex, *n* (%)	244 (36.64)	221 (37.39)	23 (30.67)	0.312
Onset to door time, min, median (IQR)	152 (120–190)	153 (120–190)	147 (114–180)	0.234
From stroke onset to thrombolysis, min, median (IQR)	190 (141.00–226.75)	190 (141.50–229.50)	181 (140.50–217.50)	0.270
NIHSS score at admission, median (IQR)	7 (4–13)	7 (4–13)	6 (4–14)	0.508
Stroke classification, *n* (%)				
Large artery atherosclerosis	277 (41.59)	242 (40.95)	35 (46.67)	0.411
Small vessel occlusion	190 (28.53)	172 (29.10)	18 (24.00)	0.432
Cardioembolism	145 (21.77)	127 (21.49)	18 (24.00)	0.728
Other determined cause	16 (2.40)	14 (2.37)	2 (2.67)	0.699
Undetermined cause	38 (5.71)	36 (6.09)	2 (2.67)	0.298
Medical history, *n* (%)				
Hypertension	414 (62.16)	368 (62.27)	46 (61.33)	0.975
Diabetes mellitus	167 (25.08)	149 (25.21)	18 (24.00)	0.931
Ischemic heart disease	146 (21.92)	130 (22.00)	16 (21.33)	1.000
Atrial fibrillation	61 (9.16)	53 (8.97)	8 (10.67)	0.789
Prior stroke/transient ischemic attack	135 (20.27)	123 (20.81)	12 (16.00)	0.410
Smoking status, *n* (%)				
Prior smoking	260 (39.04)	228 (38.58)	32 (42.67)	0.577
Current smoking	213 (31.98)	188 (31.81)	25 (33.33)	0.893
Prior medication, *n* (%)				
Antiplatelet agents	93 (13.96)	86 (14.55)	7 (9.33)	0.293
Statins	81 (12.16)	74 (12.52)	7 (9.33)	0.543
Anticoagulant agents	15 (2.25)	13 (2.20)	2 (2.67)	0.682
Antihypertension agents	294 (44.14)	261 (44.16)	33 (44.00)	1.000
Glucose lowering agents	136 (20.42)	119 (20.14)	17 (22.67)	0.719
BMI, median (IQR)	23.85 (21.64–26.07)	23.78 (21.64–26.08)	23.88 (21.65–25.83)	0.805
Blood pressure within 24 h, mmHg				
Systolic, mean ± SD	142.29 ± 16.94	140.91 ± 16.80	153.15 ± 13.82	< 0.001
Diastolic, median (IQR)	80.33 (74.33–87.00)	80.00 (74.33–86.67)	82.50 (75.33–88.54)	0.289
White blood cell count, 10^9^/L, median (IQR)	7.72 (6.12–9.63)	7.75 (6.16–9.75)	7.15 (5.92–9.11)	0.158
Neutrophil count, 10^9^/L, median (IQR)	5.26 (3.99–7.26)	5.27 (4.00–7.29)	5.15 (3.81–6.53)	0.351
Lymphocyte count, 10^9^/L, median (IQR)	1.41 (1.04–1.94)	1.42 (1.05–1.95)	1.32 (1.02–1.81)	0.322
Monocyte count, 10^9^/L, median (IQR)	0.48 (0.37–0.61)	0.49 (0.37–0.61)	0.44 (0.34–0.58)	0.118
Hemoglobin, g/L, median (IQR)	137.50 (126–147)	138.00 (126–148)	136.00 (127–146)	0.328
Platelet count, 10^9^/L, median (IQR)	191 (156.25–236.00)	193 (158.00–238.50)	184 (147.50–224.50)	0.120
Albumin, g/L, median (IQR)	39.10 (36.64–41.80)	39.10 (36.50–41.74)	39.29 (37.50–42.30)	0.202
Total cholesterol, mmol/L, median (IQR)	4.36 (3.70–5.15)	4.35 (3.69–5.12)	4.51 (3.84–5.38)	0.299
High-density lipoprotein, mmol/L, median (IQR)	1.21 (1.03–1.47)	1.20 (1.02–1.46)	1.31 (1.03–1.55)	0.162
Low-density lipoprotein, mmol/L, median (IQR)	2.62 (2.05–3.24)	2.61 (2.04–3.26)	2.62 (2.20–3.20)	0.675
Glucose, mmol/L, median (IQR)	5.94 (4.99–7.57)	5.87 (4.90–7.38)	6.91 (5.46–9.67)	0.001
Uric acid, μmol/L, median (IQR)	341.75 (268.78–399.70)	345.20 (270.50–401.35)	317.40 (248.30–375.40)	0.039
Creatinine, μmol/L, median (IQR)	74.05 (61.52–89.27)	74.40 (62.30–89.05)	69.10 (56.95–93.10)	0.301
Homocysteine, μmol/L, median (IQR)	15.04 (11.70–19.57)	15.08 (11.84–19.50)	13.92 (11.35–19.80)	0.346
Thrombolytic drug, *n* (%)				0.230
Tenecteplase	55 (8.26)	52 (8.80)	3 (4.00)	
Alteplase	611 (91.74)	539 (91.20)	72 (96.00)	
Endovascular thrombectomy performed, *n* (%)	59 (8.86)	49 (8.29)	10 (13.33)	0.218
Score of 0–2 on mRS at 90 days, *n* (%)	466 (69.97)	443 (74.96)	23 (30.67)	< 0.001
Intracranial hemorrhage within 36 h, *n* (%)				
Symptomatic	17 (2.55)	4 (0.68)	13 (17.33)	< 0.001
Asymptomatic	73 (10.96)	60 (10.15)	13 (17.33)	0.093
Moderate or severe systemic bleeding, *n* (%)	9 (1.35)	6 (1.02)	3 (4.00)	0.070

END, early neurological deterioration; IQR, interquartile range; SD, standard deviation; mRS, modified Rankin Scale; BMI, body mass index.

### 3.2 Development and performance evaluation of a nomogram for END using independent risk factors

Logistic regression models, both unadjusted and adjusted, were used to assess the relationship between risk factors and the outcome. Of the 46 baseline variables, 10 were selected for multivariable regression because they had a univariable *p*-value < 0.2 or were considered clinically meaningful (Table 2). Relative to patients without END, those who developed END had higher SBP after thrombolysis (OR 1.047, 95% CI 1.031–1.064, *p* < 0.001), elevated glucose (OR 1.094, 95% CI 1.032–1.156, *p* = 0.002), and a higher frequency of sICH (OR 30.770, 95% CI 10.529–111.882, *p* < 0.001; Table 2). Given that sICH occurs after thrombolysis and cannot serve as an early predictor, and because partial END events overlap with sICH, this variable was excluded from the multivariable regression analysis. After adjusting for potential confounders in the multivariable analysis, both SBP (OR 1.046, 95% CI 1.030–1.064, *p* < 0.001) and glucose (OR 1.079, 95% CI 1.011–1.147, *p* = 0.017) remained independently associated with END (Table 2).

**Table 2:** Binary logistic regression analysis on the association between influencing factors and END.

Variables	Univariate analysis		Multivariate analysis	
	*p* value	OR (95% CI)	*p* value	OR (95% CI)
Age, years	0.155	1.015 (0.995–1.036)	–	–
Systolic blood pressure, mmHg	< 0.001	1.047 (1.031–1.064)	< 0.001	1.046 (1.030–1.064)
White blood cell count, 10^9^/L	0.193	0.942 (0.857–1.027)	–	–
Monocyte count, 10^9^/L	0.200	0.429 (0.112–1.484)	–	–
Platelet count, 10^9^/L	0.135	0.997 (0.992–1.001)	–	–
High-density lipoprotein, mmol/L	0.221	1.466 (0.775–2.664)	–	–
Glucose, mmol/L	0.002	1.094 (1.032–1.156)	0.017	1.079 (1.011–1.147)
Uric acid, μmol/L	0.053	0.998 (0.995–1.000)	–	–
Symptomatic intracranial hemorrhage within 36 h, *n* (%)	< 0.001	30.770 (10.529–111.882)	–	–
Moderate or severe systemic bleeding within 36 h, *n* (%)	0.064	1.856 (0.930–3.479)	–	–
Endovascular thrombectomy performed, *n* (%)	0.152	1.702 (0.781–3.394)	–	–

END, early neurological deterioration; OR, odds ratio; CI, confidence interval.

The nomogram was developed from the two previously described factors (Table 2). To predict END in AIS patients receiving intravenous thrombolysis, the nomogram assigned points to each variable based on its position along the axis (Figure 1A). Second, sum the scores across all variables to obtain the total score. This composite score was then used to estimate each patient’s risk of END.

**Figure 1:**
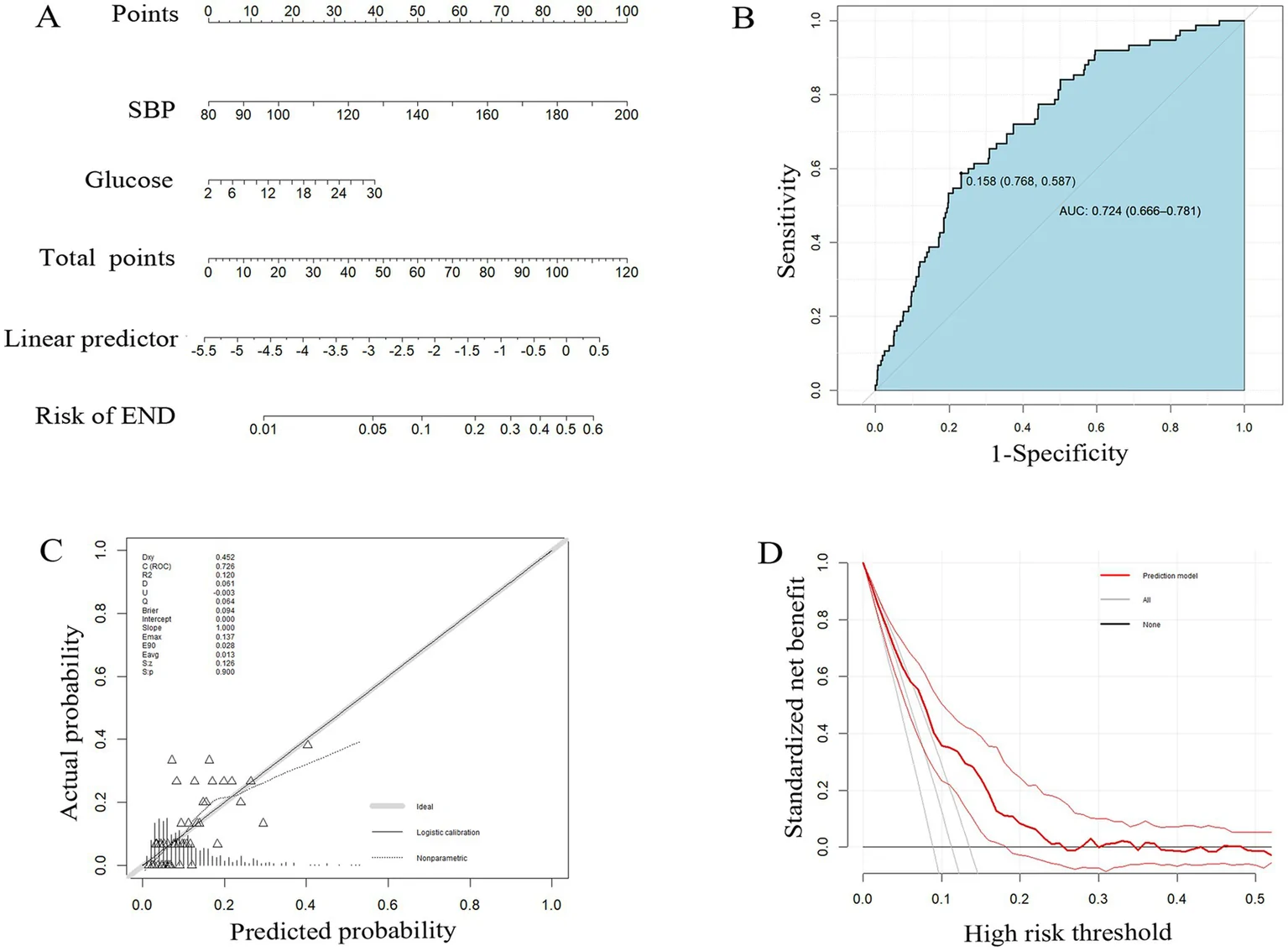
Nomogram and performance of an END prediction model in thrombolyzed stroke patients. **(A)** Nomogram for the prediction of END in stroke patients treated with intravenous thrombolysis; **(B)** The ROC curves; **(C)** The calibration curves; **(D)** The decision curve analysis. END, early neurological deterioration; SBP, systolic blood pressure; AUC, area under the curve; ROC, receiver operating characteristic.

The model showed moderate discrimination, with an AUC of 0.724 (95% CI 0.666–0.781) (Figure 1B). The optimal threshold probability of 15.8%, identified by maximizing Youden’s index, yielded a sensitivity of 58.7% and a specificity of 76.8% (Figure 1B). The calibration curve showed excellent concordance with the theoretical 1:1 line, with a slope of 1.00 and an intercept of 0.00 (Figure 1C). The Hosmer–Lemeshow analysis showed a non-significant result (*χ*^2^ = 0.016, *p* = 0.900), supporting good model fit. Furthermore, DCA demonstrated a substantially greater net benefit for the nomogram (Figure 1D). Internal cross-validation with 1,000 bootstrap replicates yielded a comparable C-index (0.7241, 95% CI 0.6647–0.7841).

### 3.3 Sensitivity analysis excluding patients with sICH

To address definitional overlap between sICH and END, we conducted a sensitivity analysis excluding all sICH patients (*n* = 17) and refitted the logistic regression model (Table 3). The sICH-free model yielded comparable results: AUC 0.719 (95% CI 0.658–0.780) vs. 0.724 (95% CI 0.666–0.781) in the primary model, with similar sensitivity (59.7% vs. 58.7%), specificity (76.5% vs. 76.8%), calibration (Hosmer-Lemeshow *p* = 0.891 vs. 0.900), and net benefit on decision curve analysis (Figure 2). Bootstrap internal evaluation also showed comparable C-indices (0.7195 vs. 0.7241). These findings suggest that our primary model’s strong performance does not depend exclusively on the overlap between sICH and END. Because the sensitivity analysis excluding sICH cases yielded results similar to the primary model, we conducted all subsequent analyses on the cohort without sICH patients to ensure consistency.

**Table 3:** Binary logistic regression analysis on the association between influencing factors and non-hemorrhagic END.

Variables	Univariate analysis		Multivariate analysis	
	*p* value	OR (95% CI)	*p* value	OR (95% CI)
Onset to door time, min	0.133	0.997 (0.993–1.001)	–	–
From stroke onset to thrombolysis, min	0.125	0.997 (0.993–1.001)	–	–
Prior stroke/transient ischemic attack, *n* (%)	0.138	0.559 (0.241–1.142)	–	–
Antiplatelet agents, *n* (%)	0.085	0.402 (0.120–1.009)	–	–
Systolic blood pressure, mmHg	< 0.001	1.043 (1.026–1.061)	< 0.001	1.040 (1.023–1.059)
Monocyte count, 10^9^/L	0.320	0.495 (0.117–1.856)	–	–
Albumin, g/L	0.369	1.031 (0.965–1.102)	–	–
Total cholesterol, mmol/L	0.130	1.198 (0.945–1.511)	–	–
High-density lipoprotein, mmol/L	0.162	1.589 (0.807–2.986)	–	–
Glucose, mmol/L	< 0.001	1.152 (1.083–1.224)	< 0.001	1.133 (1.062–1.207)
Uric acid, μmol/L	0.147	0.998 (0.995–1.001)	–	–
Creatinine, μmol/L	0.162	0.993 (0.981–1.002)	–	–
Asymptomatic ICH within 36 h, *n* (%)	0.013	2.330 (1.155–4.432)	0.143	1.710 (0.806–3.416)
Moderate or severe systemic bleeding within 36 h, *n* (%)	0.110	3.880 (0.548–18.430)	–	–
Endovascular thrombectomy performed, *n* (%)	0.078	1.997 (0.875–4.135)	–	–

END, early neurological deterioration; OR, odds ratio; CI, confidence interval; ICH, intracranial hemorrhage.

**Figure 2:**
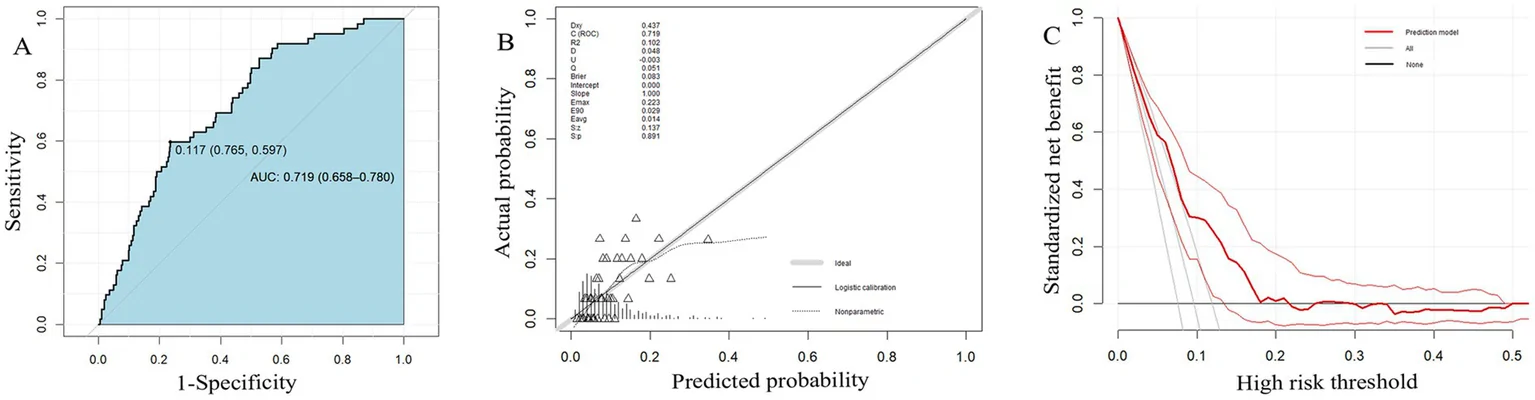
Performance of a non-hemorrhagic END prediction model in thrombolyzed stroke patients. **(A)** The ROC curves; **(B)** The calibration curves; **(C)** The decision curve analysis. END, early neurological deterioration; SBP, systolic blood pressure; AUC, area under the curve; ROC, receiver operating characteristic.

Next, we examined the predictive value of GAR (glucose/glycated hemoglobin). Because GAR strongly correlated with glucose (*r* = 0.7688, *p* < 0.0001), it could not be included in the same model. After replacing glucose with GAR and adjusting for confounders, GAR was not significantly associated with END (OR 0.999, 95% CI 0.990–1.007, *p* = 0.834). This suggests that glucose is a more informative predictor than GAR.

### 3.4 Altered gut tryptophan metabolites and inflammatory profile associated with END after thrombolysis in stroke patients

Gut microbiota dysregulation, particularly through altered tryptophan metabolism, critically mediates the gut-brain axis signaling, and inflammatory factors accelerate stroke progression. Serum levels of ILA, IPA, TMAO, MMP9, LPS, and IFN γ were measured in pre-thrombolysis serum samples from stroke patients. ELISA analysis showed lower serum concentrations of ILA and IPA in patients with END than in no-END patients (Figure 3). Conversely, END patients had elevated levels of MMP9, LPS, and IFN γ (Figure 3). Nevertheless, the expression levels of TMAO remained similar across the two groups. Furthermore, unadjusted and adjusted logistic regression models identified serum IPA, ILA, and IFN γ levels as independent factors associated with END (Table 4).

**Figure 3:**
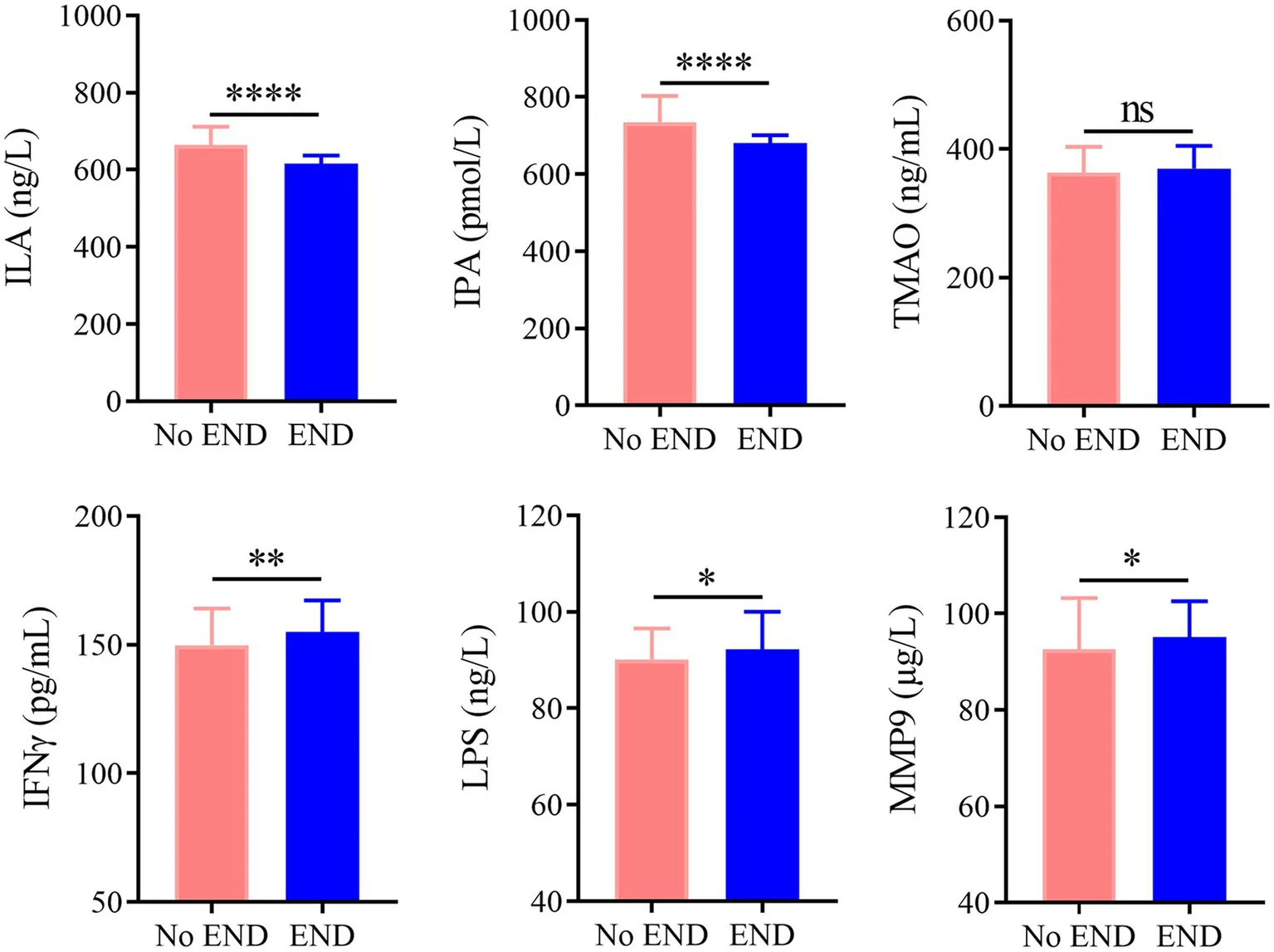
Serum levels of gut tryptophan metabolites and inflammatory cytokines were detected using ELISA in thrombolyzed stroke patients with and without END. *p < 0.05, **p < 0.01, ****p < 0.0001 (two-tailed Student’s *t*-test). ns: not significant. ILA, indole-3-lactic acid; IPA, indole-3-propionic acid; TMAO, trimethylamine N-oxide; MMP9, matrix metalloproteinase 9; LPS, lipopolysaccharide; IFN γ, interferon γ.

**Table 4:** Binary logistic analysis of gut tryptophan metabolites, inflammatory cytokines, and END.

Variables	Univariate analysis		Multivariate analysis	
	*p* value	OR (95% CI)	*p* value	OR (95% CI)
ILA, ng/L	< 0.001	0.973 (0.966–0.980)	< 0.001	0.975 (0.967–0.982)
IPA, pmol/L	< 0.001	0.987 (0.982–0.991)	< 0.001	0.988 (0.983–0.993)
TMAO, ng/mL	0.224	1.004 (0.998–1.010)	–	–
MMP9, μg/L	0.046	1.025 (1.001–1.050)	0.051	1.030 (1.000–1.062)
LPS, ng/L	0.011	1.050 (1.011–1.090)	0.118	1.034 (0.992–1.079)
IFNγ, pg./mL	0.003	1.027 (1.009–1.045)	0.003	1.052 (1.005–1.107)

ILA, indole-3-lactic acid; IPA, indole-3-propionic acid; TMAO, trimethylamine N-oxide; MMP9, matrix metalloproteinase 9; LPS, lipopolysaccharide; IFNγ, interferon γ.

### 3.5 Construction and assessment of a nomogram integrating gut tryptophan metabolites and inflammatory biomarkers

A multivariate logistic regression model was used to assess associations between risk factors and END. After controlling for potential confounders, SBP (OR 1.044, 95% CI 1.024–1.066, *p* < 0.001), glucose (OR 1.107, 95% CI 1.024–1.195, *p* = 0.009), ILA (OR 0.973, 95% CI 0.963–0.981, *P* < 0.001), IPA (OR 0.989, 95% CI 0.983–0.994, *p* < 0.001), and IFN γ (OR 1.037, 95% CI 1.012–1.064, *p* = 0.003) remained independently associated with END (Table 5). Additionally, MMP9 showed a marginally significant association (OR 1.036, 95% CI 1.001–1.075, *p* = 0.0503; Table 5).

**Table 5:** Results of multivariable regression analysis for predictors and non-hemorrhagic END.

Variables	Odds ratio (OR)	95% CI	*p* value
Systolic blood pressure	1.044	1.024–1.066	< 0.001
Glucose	1.107	1.024–1.195	0.009
ILA	0.973	0.963–0.981	< 0.001
IPA	0.989	0.983–0.994	< 0.001
MMP9	1.036	1.001–1.075	0.050
IFN γ	1.037	1.012–1.064	0.003

END, early neurological deterioration; CI, confidence interval; ILA, indole-3-lactic acid; IPA, indole-3-propionic acid; MMP9, matrix metalloproteinase 9; IFNγ, interferon γ.

The nomogram includes five predictive indicators: SBP, Glucose, ILA, IPA, and IFN γ (Figure 4A); the results confirmed each as an independent predictor of END. The model yielded strong discriminative capability, attaining an AUC of 0.909 (95% CI 0.879–0.939) (Figure 4B). The optimal cutoff probability of 13.4%, determined by maximizing Youden’s index, yielded a sensitivity of 83.9% and a specificity of 85.9% (Figure 4B). The calibration curve closely aligned with the ideal diagonal line, showing a slope of 1.000 and an intercept of 0.000 (Figure 4C). Hosmer–Lemeshow analysis showed a non-significant finding (*χ*^2^ = 0.320, *p* = 0.571), indicating good model fit. The overall model performance, evaluated using the Brier score, was 0.07, reflecting excellent predictive accuracy. Furthermore, DCA revealed a markedly superior net benefit for the nomogram (Figure 4D). Internal cross-validation employing 1,000 bootstrap replicates yielded a comparable C-index (0.905, 95% CI 0.877–0.936). This result confirms the robust reliability and stability of the prediction model in differentiating END patients from non-END patients.

**Figure 4:**
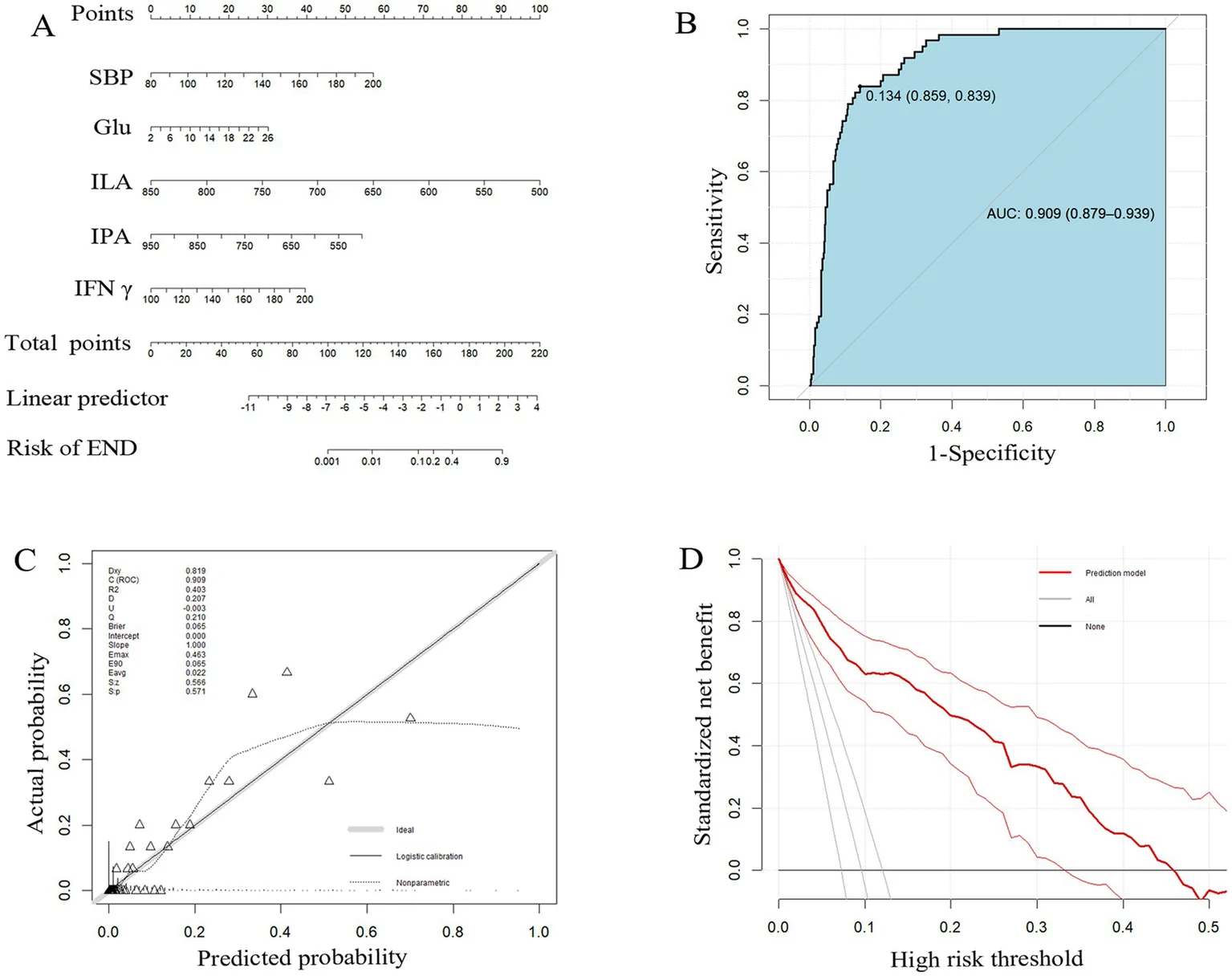
A multi-indicator nomogram incorporating gut tryptophan metabolites and inflammatory factors to predict END after thrombolysis in stroke patients. **(A)** The nomogram of the prediction model; **(B)** The ROC curves; **(C)** The calibration curves; **(D)** The decision curve analysis. SBP, systolic blood pressure; AUC, area under the curve; ROC, receiver operating characteristic.

Next, to evaluate predictive performance, we compared the base model (SBP and glucose only) with the extended model (SBP, glucose, ILA, IPA, and IFN-γ) using NRI and IDI. The categorical NRI was 0.601 (95% CI 0.477–0.725, *p* = 0.000), reflecting correct reclassification of 60.1% of individuals. The continuous NRI reached 1.369 (95% CI 1.217–1.521, *p* = 0.000), and the IDI was 0.197 (95% CI 0.162–0.233, *p* = 0.000). Together, these results confirm that adding gut tryptophan metabolites and inflammatory biomarkers meaningfully enhanced the model’s predictive accuracy.

### 3.6 Comparison of predictive performance with and without the MMP9 indicator

To address the borderline significance of MMP9 (univariate *p* = 0.046, multivariate *p* = 0.050), we performed a sensitivity analysis to compare model performance. After including MMP9, the AUC increased slightly from 0.909 to 0.912 (Figure 5A). Sensitivity remained unchanged at 83.9% in both cases, while specificity improved modestly from 85.9 to 87.7% (Figure 5A). Calibration performance was similar (Hosmer–Lemeshow test: *p* = 0.571 vs. 0.611) (Figure 5B), and the overall model performance was also comparable, with Brier scores of 0.07 and 0.06. DCA indicated a similar net benefit across clinically relevant threshold probabilities (Figure 5C). Following bootstrap internal assessment, the C-index remained the same for both models (0.905).

**Figure 5:**
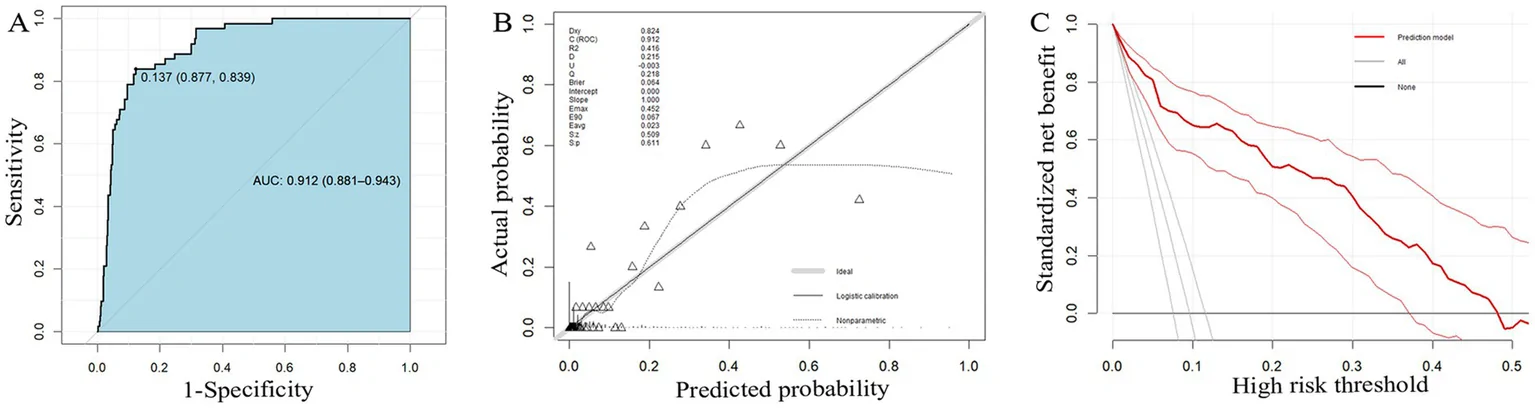
Impact of including MMP9 on the predictive accuracy of a non-hemorrhagic END model in thrombolyzed stroke patients. **(A)** The ROC curves; **(B)** The calibration curves; **(C)** The decision curve analysis.

The categorical NRI was 0.041 (95% CI − 0.060–0.142, *p* = 0.429), the continuous NRI was 0.234 (95% CI − 0.021–0.490, *p* = 0.072), and the IDI was 0.019 (95% CI 0.005–0.032, *p* = 0.006). Although the IDI was statistically significant, the actual improvement was tiny (1.86%), and the other discrimination and reclassification metrics showed no meaningful gains. Therefore, adding MMP9 did not lead to a clinically useful improvement in model performance.

## 4 Discussion

This study identified principal predictors of END following intravenous thrombolysis in stroke patients and constructed two predictive nomograms. The model integrating novel biomarkers of gut-derived tryptophan metabolites and inflammatory factors showed higher predictive accuracy (AUC = 0.909) than the conventional model (AUC = 0.719), with a categorical NRI of 0.601. Therefore, this model provides a reliable, clinically applicable approach for early identification of patients at high risk of END, thereby facilitating personalized therapeutic strategies.

END after intravenous thrombolysis remains a major clinical challenge, negatively affecting both functional recovery and long-term prognosis. In this study, we identified END as an increase of at least 4 points in the NIHSS score within 24 h following thrombolysis compared with baseline. Reported rates of post-thrombolysis END vary widely across studies. For instance, our previous research found an incidence of 19.06% (2), whereas Tian et al. reported 25.4% in a comparable cohort (3). A meta-analysis indicated an overall END rate of 11.0%, with regional differences: highest in Asia (15.9%), followed by North America (11.8%) and Europe (7.6%) (12). Such variation highlights how population characteristics and clinical practices influence outcomes. The incidence of END in our current cohort remained 11.26%, despite efforts to optimize modifiable risk factors. This persistent risk underscores the need to further investigate END predictors, particularly emerging biomarkers identified in recent years.

Gut microbiota-derived tryptophan metabolites are key mediators in regulating metabolic homeostasis and ischemic stroke pathogenesis (13). Tryptophan, an essential dietary amino acid, undergoes metabolism via three principal routes: the serotonin, kynurenine, and indole pathways. Gut microbiota metabolize indole compounds, which act as aryl hydrocarbon receptor signaling molecules to regulate immune responses and neuroinflammation, pathways implicated in stroke pathology (14, 15). IPA is an indole derivative exclusively generated by gut bacteria (16). Studies indicate that IPA exerts neuroprotective effects in cerebral ischemia (17–19). The present study found that stroke patients with END after intravenous thrombolysis exhibited a significant decrease in IPA level, which is consistent with previous research findings and even extends them (20). ILA, another indole-derived metabolite, demonstrates protective effects against ischemic stroke. Oral administration of ILA significantly mitigated brain injury following cerebral ischemia (21). Mechanistically, this protection is mediated through activation of the aryl hydrocarbon receptor, which in turn inhibits neuronal ferroptosis (22). Clinically, our findings revealed that lower circulating levels of ILA were associated with END in stroke patients after intravenous thrombolysis. These results suggest that ILA and IPA could represent novel predictive biomarkers for predicting END in stroke patients following intravenous thrombolysis. Modulating inflammatory mediators is a key neuroprotective approach following AIS. Dysbiosis of the gut microbiota can increase the release of IFN-γ from Th1 cells, which exacerbates post-stroke neuroinflammation (23). Consistent with this, the present study found that elevated IFN-γ levels independently predict END in patients receiving intravenous thrombolysis.

In this study, both SBP and glucose levels were independent predictors of END, aligning with established clinical risk profiles (2). However, the glucose result should be interpreted cautiously. Recent studies show that in patients receiving thrombolysis, the prognostic value of admission glucose is largely influenced by baseline glycemic status (24). Merlino et al. showed that stress hyperglycemia—assessed via the glucose-to-glycated hemoglobin ratio—was linked to poorer outcomes in non-diabetic patients receiving thrombolysis (24). Conversely, their 2022 study found no similar association among diabetic patients (25). To examine this, we assessed the stress hyperglycemia index in our cohort. Although admission glucose remained an independent predictor of END, the stress hyperglycemia index showed no significant association with END in multivariate analysis. This suggests that, in our cohort, the model predominantly reflects the impact of absolute hyperglycemia rather than relative glycemic elevation. To disentangle these effects, a formal stratification by diabetic status would be ideal. However, due to the small number of END events among diabetic patients, a reliable subgroup analysis was not feasible. Larger future studies are needed to confirm whether the predictive value of admission glucose differs between diabetic and non-diabetic patients receiving thrombolysis.

In the present study, three emerging serum biomarkers—ILA, IPA, and IFN γ—also showed significant predictive value for END in stroke patients after intravenous thrombolysis. While these biomarkers alone have limited diagnostic utility, combining them with conventional predictors improves risk stratification. Compared with a model without these novel markers, adding ILA, IPA, and IFN γ improved END prediction in thrombolysed patients. This integrated approach may help clinicians personalize treatment and provide more informed prognostic guidance to patients and families. To further assess the added value of our nomogram, we compared it with two representative END prediction models from the literature (model A by Wei et al., 2025 (5) and model B by Fang et al., 2025 (1)), both of which target similar outcomes and use comparable variables. Our nomogram showed stronger discriminative performance, with an AUC of 0.909 (95% CI: 0.879–0.939), outperforming model A (0.740, 95% CI: 0.686–0.789) and model B (0.809, 95% CI: 0.732–0.885). Furthermore, both the continuous NRI (1.369, 95% CI: 1.217–1.521) and IDI (0.197, *p* = 0.000) indicated significant improvements in reclassification and discrimination. DCA also showed that our model offered greater net benefit across most thresholds, supporting its clinical value for early risk stratification. We recognize that direct comparisons have limitations due to differences in study populations and outcome definitions. Despite this, our nomogram consistently outperformed others across multiple metrics and cohorts, confirming its added predictive value.

Our findings are directly relevant to clinical decision-making. While conventional risk factors such as blood pressure and glucose can be addressed with standard treatment protocols, the newly identified biomarkers—ILA, IPA, and IFN γ—enable risk assessment that goes beyond basic vital signs. For patients with elevated levels of these inflammatory markers, intensified neurological monitoring—such as NIHSS assessments every 1–2 h during the first 24–48 h—may be beneficial in a stroke unit or intermediate care setting, given their increased risk of subclinical deterioration. Although this is a high-risk population, careful blood pressure management (emphasizing stability rather than achieving rapid reduction) and glucose control (to avoid both hyperglycemia and hypoglycemia) are essential, as their effects may be heightened in the presence of inflammation. The link between IFN γ (a key pro-inflammatory cytokine), IPA, and ILA with END suggests that early anti-inflammatory or immunomodulatory treatment may be beneficial. Future interventional studies could examine whether treatments such as statins (to reduce inflammation) or specific cytokine inhibitors may reduce the risk of END in this biomarker-defined subgroup.

Several limitations should be acknowledged in the present investigation. First, as the population was primarily recruited from a single center, the lack of external validation may affect the model’s generalizability. Although the results were derived exclusively from internal assessment, a threefold evidence chain comprising bootstrap optimistic adjustment, DCA, and NRI provided convincing justification. Second, definitional overlap between sICH and END raised a major confounding concern. We addressed this with a sensitivity analysis that excluded all sICH patients from the cohort. The model’s performance remained stable, indicating that the key findings are not solely due to this overlap, and that it identifies additional END determinants beyond sICH. Nevertheless, we acknowledge that excluding sICH patients could reduce sample size and introduce selection bias, and residual confounding from unmeasured factors remains possible. Larger prospective cohorts are needed to confirm the distinct predictive roles of END subtypes and clarify how sICH interacts with other END pathways. Third, ILA and IPA were measured using commercial ELISA kits rather than liquid chromatography–tandem mass spectrometry, the reference method for quantifying small molecules. The absence of in-house assessment data—such as spike recovery and intra-assay coefficients of variation—may reduce the accuracy of absolute concentrations. However, relative group differences remained robust in regression analyses. Future studies should employ liquid chromatography–tandem mass spectrometry or fully validated ELISA kits to verify these results. Fourth, the selected biomarkers and clinical variables, though informative, may not encompass all relevant prognostic factors. Fifth, subgroup analyses of long-term outcomes related to END were not conducted due to sample size constraints. Despite these limitations, the model effectively integrates emerging serum biomarkers using rigorous methodology, providing a concise yet robust tool with strong predictive accuracy.

## 5 Conclusion

In this study, we identified several gut tryptophan metabolites and inflammatory biomarkers as predictors of END. Nomogram models incorporating these predictors demonstrated high predictive accuracy, offering potential guidance for personalized therapeutic strategies. The model will be further tested using datasets from multiple external institutions to better assess its real-world applicability.

## Data Availability

The original contributions presented in the study are included in the article/Supplementary material, further inquiries can be directed to the corresponding author/s.
