# Supplementary material for: Gut microbiota-derived tryptophan metabolites and inflammatory factors enhance prediction of early neurological deterioration after intravenous thrombolysis in stroke patients: a comparative model study
Source: Front Neurol. 2026 Oct 20;17:1934189. doi: 10.3389/fneur.2026.1934189 (PMC13727748; doi:10.3389/fneur.2026.1934189)
Supplement: Supplementary file 1 [file Supplementary_file_1.docx]

Supplementary Table S1 Comparisons of baseline characteristics between participants with and without excellent prognosis

Variables	Total (*n* = 75)	Unfavorable (*n* = 52)	Favorable (*n* = 23)	*P* value
Age, years, median (IQR)	72 (61_79)	75.5 (66–81)	68 (58–73.5)	0.013
Male sex, *n* (%)	52 (69.33)	36 (69.23)	16 (69.57)	1.000
Onset to door time, min, median (IQR)	147 (114–180)	162.5 (117–182)	133 (112–180)	0.568
From stroke onset to thrombolysis, min, median (IQR)	181 (140.50–217.50)	181.5 (140.75–227.00)	173 (137.50–201.50)	0.411
NIHSS score at 24h, median (IQR)	14 (9–21)	17.5 (11.75–22.25)	9 (7.5–10.5)	< 0.001
Stroke classification, *n* (%)				
Large artery atherosclerosis	35 (46.67)	25 (48.08)	10 (43.48)	0.907
Small vessel occlusion	18 (24.00)	7 (13.46)	11 (47.83)	0.004
Cardioembolism	18 (24.00)	16 (30.77)	2 (8.70)	0.077
Other determined cause	2 (2.67)	2 (3.85)	0 (0.00)	1.000
Undetermined cause	2 (2.67)	2 (3.85)	0 (0.00)	1.000
Occlusion site, *n* (%)				0.174
Internal carotid artery	13 (27.08)	11 (29.73)	2 (18.18)	
First segment of the middle cerebral artery	17 (35.42)	14 (37.84)	3 (27.27)	
Second segment of the middle cerebral artery	10 (20.83)	7 (18.92)	3 (27.27)	
Vertebral artery	1 (2.08)	1 (2.70)	0 (0.00)	
Basilar artery	3 (6.25)	3 (8.11)	0 (0.00)	
Posterior cerebral artery	2 (4.17)	0 (0.00)	2 (18.18)	
Anterior cerebral artery	2 (4.17)	1 (2.70)	1 (9.09)	
Medical history, *n* (%)				
Hypertension	46 (61.33)	31 (59.62)	15 (65.22)	0.840
Diabetes mellitus	18 (24.00)	15 (28.85)	3 (13.04)	0.236
Ischemic heart disease	16 (21.33)	13 (25.00)	3 (13.04)	0.362
Atrial fibrillation	8 (10.67)	7 (13.46)	1 (4.35)	0.422
Prior stroke/TIA	12 (16.00)	9 (17.31)	3 (13.04)	0.745
Smoking status, *n* (%)				
Prior smoking	32 (42.67)	20 (38.46)	12 (52.17)	0.393
Current smoking	25 (33.33)	16 (30.77)	9 (39.13)	0.658
Prior medication, *n* (%)				
Antiplatelet agents	7 (9.33)	5 (9.62)	2 (8.70)	1.000
Statins	7 (9.33)	5 (9.62)	2 (8.70)	1.000
Anticoagulant agents	2 (2.67)	2 (3.85)	0 (0.00)	1.000
Antihypertension agents	33 (44.00)	23 (44.23)	10 (43.48)	1.000
Glucose lowering agents	17 (22.67)	14 (26.92)	3 (13.04)	0.305
BMI, mean ± SD	23.63 ± 3.12	23.39 ± 2.93	24.16 ± 3.54	0.365
Blood pressure within 24 hr, mmHg				
Systolic, mean ± SD	153.15 ± 13.82	155.03 ± 13.56	148.92 ± 13.77	0.083
Diastolic, mean ± SD	81.50 ± 9.70	82.50 ± 10.00	79.24 ± 8.77	0.161
White blood cell count, 10^9/L, median (IQR)	7.15 (5.92–9.11)	7.46 (6.08–9.15)	7.04 (5.18–8.46)	0.181
Neutrophil count, 10^9/L, median (IQR)	5.15 (3.81–6.53)	5.28 (4.39–7.43)	4.31 (2.85–5.99)	0.012
Lymphocyte count, 10^9/L, median (IQR)	1.32 (1.02–1.81)	1.24 (0.94–1.58)	1.75 (1.27–2.06)	0.009
Monocyte count, 10^9/L, median (IQR)	0.44 (0.34–0.58)	0.44 (0.35–0.63)	0.42 (0.34–0.52)	0.531
Hemoglobin, g/L, mean ± SD	135.11 ± 15.42	132.85 ± 14.60	140.22 ± 16.32	0.070
Platelet count, 10^9/L, mean ± SD	189.35 ± 59.40	189.25 ± 57.41	189.57 ± 65.00	0.984
Albumin, g/L, median (IQR)	39.29 (37.50–42.30)	38.85 (37.50–41.95)	40.4 (37.50–42.95)	0.425
Total cholesterol, mmol/L, mean ± SD	4.58 ± 1.03	4.51 ± 1.02	4.76 ± 1.06	0.345
High-density lipoprotein, mmol/L, median (IQR)	1.31 (1.03–1.55)	1.33 (1.03–1.56)	1.30 (1.00–1.49)	0.411
Low-density lipoprotein, mmol/L, mean ± SD	2.75 ± 0.85	2.66 ± 0.86	2.96 ± 0.80	0.146
Glucose, mmol/L, median (IQR)	6.91 (5.46–9.67)	7.38 (5.70–10.03)	5.98 (5.19–7.74)	0.177
Uric acid, µmol/L, mean ± SD	322.02 ± 101.05	326.00 ± 109.05	313.03 ± 81.62	0.571
Creatinine, µmol/L, median (IQR)	69.10 (56.95–93.10)	75.40 (62.85–97.95)	59.20 (53.10–72.95)	0.009
Homocysteine, µmol/L, median (IQR)	13.92 (11.35–19.80)	14.74 (11.38–20.65)	13.70 (10.95–17.58)	0.186
ILA, ng/L, median (IQR)	610.14 (602.70–622.73)	611.17 (602.86–623.80)	608.11 (601.77–619.52)	0.425
IPA, pmol/L, mean ± SD	680.92 ± 20.29	680.48 ± 20.70	681.93 ± 19.72	0.774
TMAO, ng/mL, mean ± SD	369.69 ± 35.44	368.24 ± 31.07	372.95 ± 44.38	0.648
MMP9, µg/L, mean ± SD	95.16 ± 7.42	95.52 ± 7.06	94.33 ± 8.29	0.553
LPS, ng/L, median (IQR)	91.35 (88.77–96.77)	90.98 (88.30–97.39)	92.37 (89.72–95.29)	0.516
IFNγ, pg/mL, mean ± SD	154.97 ± 12.25	154.47 ± 13.24	156.09 ± 9.82	0.557
Thrombolytic drug, *n* (%)				0.548
Tenecteplase	3 (4.00)	3 (5.77)	0 (0.00)	
Alteplase	72 (96.00)	49 (94.23)	23 (100.00)	
Endovascular thrombectomy performed, *n* (%)	10 (13.33)	9 (17.31)	1 (4.35)	0.162
Symptomatic intracranial hemorrhage, *n* (%)	13 (17.33)	13 (25.00)	0 (0.00)	0.007
Asymptomatic intracranial hemorrhage, *n* (%)	13 (17.33)	10 (19.23)	3 (13.04)	0.743
Moderate or severe systemic bleeding, *n* (%)	3 (4.00)	3 (5.77)	0 (0.00)	0.548
Tirofiban, *n* (%)	30 (40.00)	14 (26.92)	16 (69.57)	0.001

END, early neurological deterioration; IQR, interquartile range; SD, standard deviation; mRS, modified Rankin Scale; BMI, body mass index; ILA, indole-3-lactic acid; IPA, indole-3-propionic acid; TMAO, trimethylamine N-oxide; MMP9, matrix metalloproteinase 9; LPS, lipopolysaccharide; IFNγ,

Supplementary Table S2 Multivariate analysis of good prognosis in the moderate NIHSS score subgroup

Variables	OR (95% CI)	*P* value
Tirofiban, *n* (%)	1.003 (0.076–11.571)	0.998
Age, years	1.012 (0.931–1.104)	0.757
Small vessel occlusion, *n* (%)	2.816 (0.514–19.871)	0.235
Neutrophil count, 10^9/L	0.528 (0.123–0.896)	0.008
Lymphocyte count, 10^9/L	1.794 (0.825–8.569)	0.162
Creatinine, µmol/L	0.985 (0.918–1.042)	0.600
